# The effects of whey, pea, and collagen protein supplementation beyond the recommended dietary allowance on integrated myofibrillar protein synthetic rates in older males: a randomized controlled trial

**DOI:** 10.1016/j.ajcnut.2024.05.009

**Published:** 2024-05-16

**Authors:** James McKendry, Caroline V Lowisz, Arraksana Nanthakumar, Meaghan MacDonald, Changhyun Lim, Brad S Currier, Stuart M Phillips

**Affiliations:** 1 Department of Kinesiology, Exercise Metabolism Research Group, McMaster University, Hamilton, Ontario, Canada; 2 School of Human Kinetics, Faculty of Health Sciences, University of Ottawa, Ottawa, Ontario, Canada

**Keywords:** sarcopenia, aging, muscle anabolism, stable isotope tracers, nutrient requirements, protein nutrition, plant-based protein

## Abstract

**Background:**

Skeletal muscle mass is determined predominantly by feeding-induced and activity-induced fluctuations in muscle protein synthesis (MPS). Older individuals display a diminished MPS response to protein ingestion, referred to as age-related anabolic resistance, which contributes to the progression of age-related muscle loss known as sarcopenia.

**Objectives:**

We aimed to determine the impact of consuming higher-quality compared with lower-quality protein supplements above the recommended dietary allowance (RDA) on integrated MPS rates. We hypothesized that increasing total protein intake above the RDA, regardless of the source, would support higher integrated rates of myofibrillar protein synthesis.

**Methods:**

Thirty-one healthy older males (72 ± 4 y) consumed a controlled diet with protein intake set at the RDA: control phase (days 1–7). In a double-blind, randomized controlled fashion, participants were assigned to consume an additional 50 g (2 × 25g) of whey (*n* = 10), pea (*n* = 11), or collagen (*n* = 10) protein each day (25 g at breakfast and lunch) during the supplemental phase (days 8–15). Deuterated water ingestion and muscle biopsies assessed integrated MPS and acute anabolic signaling. Postprandial blood samples were collected to determine feeding-induced aminoacidemia.

**Results:**

Integrated MPS was increased during supplemental with whey (1.59 ± 0.11 %/d; *P* < 0.001) and pea (1.59 ± 0.14 %/d; *P* < 0.001) when compared with RDA (1.46 ± 0.09 %/d for the whey group; 1.46 ± 0.10 %/d for the pea group); however, it remained unchanged with collagen. Supplemental protein was sufficient to overcome anabolic signaling deficits (mTORC1 and rpS6), corroborating the greater postprandial aminoacidemia.

**Conclusions:**

Our findings demonstrate that supplemental protein provided at breakfast and lunch over the current RDA enhanced anabolic signaling and integrated MPS in older males; however, the source of additional protein may be an important consideration in overcoming age-related anabolic resistance.

This trial was registered clinicaltrials.gov as NCT04026607.

## Introduction

Advances in health care provision has given rise to unprecedented population aging. Concerningly, the healthspan—the proportion of life spent in good health—has failed to increase in parallel with lifespan [1]. A decline in physical mobility and health is due, at least in part, to the insidious loss of skeletal muscle mass and function, known as sarcopenia [2,3]. Sarcopenia increases risk of mobility limitations, falls, fractures, hospitalization, and frailty [4,5]. If sarcopenia remains unchecked, older individuals could cross a threshold of physical dependence sooner, relying on care providers, and their quality of life may be considerably worsened. Interventions targeting sarcopenia may help extend healthspan and promote physical mobility and thus should be a primary focus of geroscience [1].

Although muscle protein synthesis (MPS) and muscle protein breakdown (MPB) are modifiable processes, MPS is more sensitive to the influence of nutritional and activity-related stimuli [6,7]. Protein feeding stimulates MPS robustly in young, healthy adults [8]. However, the muscles of older individuals exhibit a diminished capacity to increase MPS in response to ordinarily anabolic stimuli such as protein feeding and resistance exercise [9], a phenomenon known as age-related muscle anabolic resistance. The magnitude of the MPS impairment in response to protein/amino acid (AA) feeding may be as large as ∼30% [10].

The United States–Canadian dietary guidelines recommended dietary allowance (RDA) for protein intake remains the same for all adults (0.8 g/kg/d), regardless of age, with little reference to source or pattern of intake [11]. Several factors have been proposed to affect older adults’ MPS response, including quantity (per-meal protein “dose”) [8], quality [12], and distribution [13] of protein intake. MPS is a graded, saturable process, and the stimulation of MPS is directly related to the protein dose and subsequent aminoacidemia, reliant on essential amino acids (EAAs) and, in particular, leucine. Older individuals may require ≤67% more protein per-meal to elicit the same acute muscle anabolic response as their younger counterparts [9], and consensus statements support an RDA that is ∼50% greater than current guidelines (1.2 g/kg/d) [[14], [15], [16]].

Animal-based protein sources are considered higher-quality than plant-derived protein sources owing to their AA profile [17], stimulating MPS more effectively [18]. However, mitigating plant-derived proteins’ inferior capacity to stimulate MPS is feasible through complementary and isolated protein sources [19]; however, limited data exist in older populations.

Protein intake throughout the day (i.e., distribution) in older individuals is often skewed toward 1 meal; typically, this is the last (dinner) meal of the day in the United States and Canada [20]. Recent evidence suggests that even in individuals consuming sufficient protein, redistributing protein intake more evenly throughout the day and targeting lower protein-containing meals may help preserve muscle mass in older individuals [21].

The primary aim of this study was to investigate the impact of consuming higher-quality compared with lower-quality protein supplements (at breakfast and lunchtime meals), added to a weight-maintaining diet compared with a diet with protein at the RDA, on the integrated rates of MPS in older males. We hypothesized that compared with the period of consumption with protein at the RDA (control phase), when supplemented (supplemental phase), MPS would be enhanced in the whey, pea, and collagen protein supplement–consuming groups.

## Methods

### Statement of ethics

This study was approved by the Hamilton Integrated Research Ethics Board (HIREB) and conformed to the standards for the use of human subjects in research as outlined by the Canadian Tri-Council Policy Statement: Ethical Conduct for Research Involving Humans—TCPS 2, 2022 (https://ethics.gc.ca/eng/policy-politique_tcps2-eptc2_2022.html). Each participant was informed of the purpose of the study and experimental procedures, and potential risks before written informed consent was obtained. The trial was registered on http://www.clinicaltrials.gov as NCT04026607 and occurred at McMaster University.

### Participants

Thirty-one generally healthy, older (72 ± 4 y) males were recruited from the local Hamilton, Ontario, area via public advertisements, social media, and a local radio broadcast. Participants were recruited between January 2020 and March 2020, before the study was interrupted by restrictions on human research testing owing to the COVID-19 pandemic; several participants had to withdraw from the study during this time and were unavailable for retesting. Participant recruitment was resumed in March 2022, and data collection was completed by December 2022. Participants were deemed eligible to participate if they were males aged 65–80 y (inclusive), nonsmokers, and in good general health, as determined by a medical screening questionnaire. Participants were excluded for the following reasons: *1*) if they experienced an orthopedic, cardiovascular, pulmonary, renal, liver, infectious disease, immune, metabolic, or gastrointestinal disorder, which are likely to impact study outcomes; *2*) if they took medications known to affect protein metabolism (i.e., corticosteroids, nonsteroidal anti-inflammatory drugs, high strength acne medication, or testosterone replacement); *3*) if they used tobacco or tobacco-related products; *4*) if they consumed a vegan or vegetarian diet; *5*) if they required an assistive walking device; or *6*) if they already consumed or had a sensitivity to the ingredients being tested [e.g., protein/branched-chain amino acid (BCAA) supplements].

### Sample size power calculation

Based on our previous work [[22], [23], [24]], using G∗power (version 3.1.9.6), we determined that 12 participants per group would be sufficient to attain 80% power and α at 0.05. With an expected dropout rate of ∼15%, we sought to recruit 15 participants per group. A total sample size of 45 would ensure we were adequately powered to detect a 10% difference in integrated MPS (i.e., the hypothesized difference between whey based on variance from past measurements and using an α of 0.05).

### Experimental design and study overview

**FIGURE 1:**
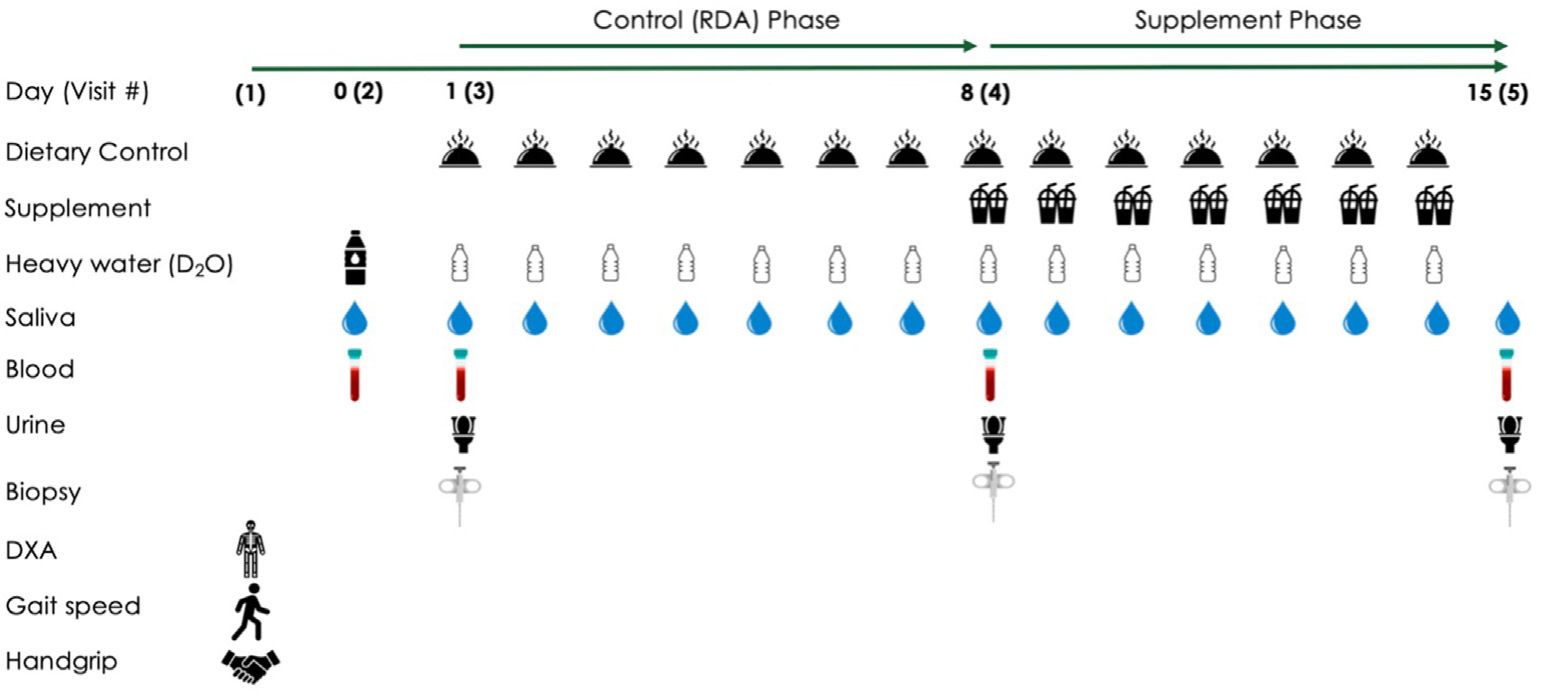
Schematic overview of the study design. DXA, dual X-ray absorptiometry.

In a double-blind, randomized parallel-group trial, participants were randomly assigned to 1 of the 3 groups: whey, pea, or collagen, using a block concealed allotment procedure (equal chance) based on the order in which they were screened for participation. The randomization sequence was generated using https://www.sealedenvelope.com/simple-randomiser/v1/lists. Following screening and baseline testing, participants completed two 7-d study phases in sequence: *1*) control phase—all participants consumed a standardized diet with protein intake fixed at the RDA (0.8 g/kg/d), and *2*) supplemental phase—participants consumed the standardized diet, with the addition of twice daily protein beverage supplementation (i.e., 2 × 25 g) from 1 of the 3 protein supplements (whey: Sureprotein Instant WPC450; New Zealand Milk Products; pea: NUTRALYS S85 Plus N; Roquette Frères; or collagen: Gelita Bodybalance; Gelita). Our primary outcome was integrated myofibrillar protein synthesis, and our secondary assessments were acute anabolic signaling and blood aminocidemia. A schematic overview of the study protocol is shown in Figure 1.

### Screening visit (visit 1)

Participants arrived at the laboratory in an overnight fasted state. Participants completed a general health questionnaire to indicate their current health status, medication use, and any dietary restrictions, ensuring eligibility for the study. During the screening visit, height and body mass were assessed using a calibrated stadiometer and scale. Afterward, dressed in loose-fitting athletic clothing, participants underwent a dual X-ray absorptiometry (GE-Lunar iDXA; Aymes Medical) scan to assess body composition, including lean mass (LM), appendicular LM, and total fat mass. Participants also performed gait speed (6-min) and handgrip strength assessments (Jamar Plus+ Digital Hand Dynamometer) to determine physical function and muscular strength, respectively. Following the screening visit, participants were given a 3-d weighed-food diet log to record their habitual dietary intake for 2 weekdays and 1 weekend day between screening and study commencement [[25], [26], [27]]—to assess habitual energy intake (NUTRIUM; Braga) and dietary preferences to inform the controlled study diet.

### Deuterated water loading (visit 2)

Following an overnight fast, participants arrived at the laboratory (∼08:00) and provided a baseline (i.e., unenriched) saliva and blood sample before undergoing the heavy water loading. To achieve a ^2^H enrichment of the body water pool at ∼0.4–0.5 atom percent excess (APE) and minimize the likelihood of side effects, participants consumed 8 doses of deuterated water (70 atom%; Cambridge Isotope Laboratories) at 60- to 90-min intervals throughout the day. Each dose of deuterated water was equivalent to 0.65 mL/kg dual X-ray absorptiometry–derived LM. Participants consumed 3 doses in the investigator’s presence and the remaining doses on leaving the laboratory. Participants were also given salivettes to collect saliva samples on waking each morning for ^2^H enrichment analysis and topup doses of deuterated water (D_2_O) to consume after the saliva sample to maintain the body water enrichment. On leaving, participants were provided wrist-worn accelerometers to monitor physical activity throughout the study.

### Main study visits (3, 4, and 5)

After an overnight fast, participants arrived at the laboratory, and a catheter was placed in their antecubital vein for repeated blood sampling. Participants provided basal saliva, urine, and blood samples. Next, participants were fed a standardized, prepackaged breakfast consisting of scrambled eggs with mixed vegetables and hash brown potatoes [320 kcal, 21 g carbohydrate (5g sugar), 19 g fat, and 16 g protein; Heart to Home; Kitchener) and a juice box [80 kcal, 21 g carbohydrate (19 g sugar), 0 g fat, and 0 g protein to be consumed within ∼15 min]. Following breakfast, blood samples were collected every 15 min for 1 h, and a skeletal muscle biopsy was collected 60 min postbreakfast.

During the control phase, participants were given a standardized diet consisting of 3 primary prepackaged meals (breakfast, lunch, and dinner; Heart to Home, Kitchener) and a mix of fruits, vegetables, snacks, and drinks. Participants were instructed to maintain their habitual physical activity levels.

Following the control phase, participants returned (fasted) for the same acute feeding visit; however, for this visit, the participants were fed the same standardized breakfast plus a single serving (25 g) of the protein supplement in line with the group to which they were assigned—whey, pea, or collagen. Again, basal saliva, urine, and blood samples were collected, followed by repeated blood samples for 1 h postfeeding. A skeletal muscle biopsy was collected 1 h postfeeding, and participants were sent home with the same standardized diet and the protein supplements to be consumed twice daily (supplemental phase) for the next 7 d. Following the 7-d supplemental phase, participants returned to the laboratory and provided a final resting-fasted saliva, urine, blood, and muscle biopsy sample.

### Dietary control and physical activity monitoring

Daily energy requirements were determined using the Harris–Benedict equation, factoring in physical activity levels; resting energy expenditure was multiplied by 1.375 to account for the light-to-moderate levels of physical activity typically performed by older individuals not performing structured exercise training. During the control phase, standardized diets provided protein at the RDA (0.8 g/kg body mass/d), distributed unevenly (i.e., most of the protein was ingested at dinner) in line with the typical eating patterns of older adults in Canada and the United States [28]. The energy composition of the standardized diet provided was designed to mimic the habitual dietary patterns of older individuals (i.e., ∼55% carbohydrate, ∼30% fat, and ∼15% protein). During the supplemental phase, participants consumed the same standardized diet with a 25-g protein supplement at breakfast and lunch—totaling an additional 50 g each day and a more even protein distribution. Adherence to the diet was confirmed verbally by participants, and supplement consumption was assessed by having participants return the empty supplement sachets. Participants were asked to refrain from intense exercise but maintain habitual physical activity throughout the protocol. Throughout the study (from visits 2 to 5), participants’ physical activity and step count were monitored using an accelerometer (Actigraph wGT2X-BT activity monitor; ActiGraph) on the wrist of their nondominant arm.

### Nutritional supplements

The details for each protein supplement provided in this study can be found in Table 1. Individual servings of each powdered supplement were matched for appearance and flavor and prepared in identical packaging (blinded) by Gruppo Nutrition. Participants were given a protein shaker and 14 × 25 g servings (1 of which was consumed during visit 4 in our laboratory) of the supplement and instructed to mix each package with 400 mL water and shake vigorously before ingestion.

**TABLE 1:** Nutritional supplement information

	Collagen (Bodybalance)		Whey (Sureprotein WPC450)		Pea (NUTRALYS S85 PLUS N)	
	Per 100 g	Per 25 g protein	Per 100 g	Per 25 g protein	Per 100 g	Per 25 g protein
Micronutrients						
Energy (kcal)	393	116	402	126	400	127
Fat (g)	0.6	0.2	6.0	1.9	9.0	2.9
Carbohydrate (g)	3.7	1.1	6.1	1.9	0	0
Sugar (g)	0.7	0.2	6.1	1.9	0	0
Fiber (g)	0.9	0.3	0	0	1.0	0.3
Protein (g)	84.6	25	79.8	25	79.0	25
Amino acids						
Aspartic acid	5.50	1.63	8.90	2.80	9.13	2.89
Glutamic acid	9.80	2.90	14.50	4.50	13.26	4.20
Alanine	9.50	2.81	4.30	1.30	3.41	1.08
Arginine	7.60	2.25	1.90	0.60	6.91	2.19
Cysteine	0.08	0.02	2.00	0.60	0.79	0.25
Glycine	23.20	6.86	1.50	0.50	3.18	1.01
Histidine	0.50	0.15	1.40	0.40	1.99	0.63
Isoleucine	1.70	0.50	5.20	1.60	3.73	1.18
Leucine	2.90	0.86	8.70	2.70	6.51	2.06
Lysine	3.60	1.06	7.50	2.30	5.64	1.78
Methionine	0.80	0.24	1.80	0.60	0.87	0.28
Phenylalanine	1.70	0.50	2.60	0.80	4.37	1.38
Proline	12.80	3.78	5.00	1.60	3.41	1.08
Serine	3.20	0.95	4.40	1.40	4.05	1.28
Threonine	2.0	0.59	6.40	2.00	3.02	0.96
Tyrosine	0.40	0.12	2.60	0.80	3.02	0.96
Valine	2.30	0.68	4.70	1.50	3.97	1.26
Tryptophan	0.01	0.00	1.60	0.50	0.79	0.25
∑EAAs	15.51	4.58	39.90	12.50	30.89	9.78
∑NEAAs	72.08	21.3	45.10	14.10	47.16	14.92

Abbreviations: EAA, essential amino acid; NEAA, nonessential amino acid.

### Muscle biopsies

All muscle biopsies were taken from the vastus lateralis following administration of local anesthesia (1% xylocaine with epinephrine) with the use of a 5-mm Bergström needle custom-modified for manual suction. Muscle tissue samples were freed from any visible connective and adipose tissue, rapidly frozen in liquid nitrogen, and stored at −80°C until later analysis. The initial leg for biopsy was randomly determined, and the leg was alternated for subsequent biopsies (i.e., right, left, and right or vice versa).

### Integrated myofibrillar protein synthesis

Consumption of D_2_O was used to label newly synthesized myofibrillar proteins [29]. MPS rates were determined using the standard precursor–product method, as we have described previously [30,31]. Total body water (saliva) deuterium (^2^H) enrichment was used as a surrogate for plasma alanine labeling (precursor). The change in ^2^H enrichment (relative to ^1^H) of muscle alanine (product) over time was used to calculate the myofibrillar fractional synthesis rates for the control and supplemental phases:

$$\text{FSR}\;\left(\%/d\right)=\left[\frac{\left(E_{\text{Ala}2}-E_{\text{Ala}1}\right)}{E_{\text{BW}}\;\cdot \;t}\right]\cdot \;3.7\;\cdot \;100$$

*E*_Ala*x*_ is the APE of protein-bound enrichment from muscle biopsies at *x* times 1 and 2 (Figure 1 for protocol). *E*_BW_ is the mean ^2^H enrichment (in APE) of total body water between the time points. Finally, *t* is the tracer incorporation time in days (i.e., 7). Multiplication by 3.7 adjusts for the mean number of ^2^H atoms that can become incorporated into alanine [29], and multiplication by 100 converts the values to percentages.

### Analyses

Muscle samples (∼30 mg) were homogenized using 5-mm stainless steel beads in a 2-mL Eppendorf (2 × 40 s at 20 Hz; TissueLyser) with 500 μL fresh ice-cold homogenization buffer [25 mM Tris buffer; Tris-HCl, Trizma Base, double-distilled H_2_O (ddH_2_O), pH 7.2], 1 PhosStop Tablet (Roche), 1 complete (Roche) miniprotease inhibitor tab, 100 μL TritonX-100). Samples were centrifuged at 2264 g for 10 min at 4°C to separate the sarcoplasmic (supernatant used for western blotting analysis) and myofibrillar fractions. The myofibrillar fraction was purified by adding 500 μL of ddH_2_O, vortexing for 5 s and centrifuging at 252 g for 10 min at 4°C. Next, 1 mL of 0.3 M NaOH was added to the sample and vortexed before being placed in a heating block at 50°C for 30 min (vortex 5 s every 10 min) to solubilize the myofibrillar proteins. Samples were centrifuged at 11180 g for 10 min at 4°C to pellet the collagen proteins, and the supernatant (containing the myofibrillar fraction) was placed in a 4-mL glass screw-top tube. Proteins were precipitated with 1 mL of 1 M perchloric acid and centrifuged at 1363 g for 10 min at 4°C. After removing the supernatant, the myofibrillar protein pellet was washed twice in 70% ethanol (centrifuging at 1363 g for 10 min at 4°C). AAs were liberated by adding 1 mL of Dowex resin (50WX8-100-200 mesh resin; Sigma-Aldrich) and 1 mL of 1 M HCL before heating at 110°C for 72 h. The free AAs were purified on cation-exchange columns, dried under vacuum in a rotary evaporator, and reconstituted in 0.1 M HCl before gas chromatography-pyrolysis-isotope ratio mass spectrometry analysis. Resulting AA preparations were analyzed for deuterated-alanine (^2^H-alanine) with a Thermo Finnigan Delta V isotope ratio mass spectrometry coupled to a Thermo Trace GC Ultra with a gas chromatography combustion interface III and Conflow IV. The N-acetyl n-propyl ester of alanine was analyzed using a splitless injection and a Zebron ZB-5 column of 30 m × 0.25 mm × 0.50 μm film thickness (Phenomenex). The gas chromatography oven was programmed with an initial column temperature of 80°C with a 2-min hold, followed by a ramp of 30°C/min to 330°C. Eluents were directed into the pyrolysis reactor, heated at 1450°C, and converted to hydrogen gas (Metabolic Solutions).

### Body water ^2^H enrichment

Saliva samples were analyzed for ^2^H enrichment by cavity ringdown spectroscopy (L2130-I; Picarro). Briefly, the ^2^H-enriched saliva samples were diluted (1:50) with double-distilled water and analyzed using express mode (i.e., injected 10 times: 6 wet flushes and 4 sample injections) with the mean of the last 3 measurements used for analysis. Measurements were correct for machine drift and background enrichment, and the ^2^H (D) isotopic enrichments for saliva were converted to APE using the following standard equation:

$$\text{APE}=\left[\frac{100\;\cdot \;\text{AR}\;\cdot \;\left(\delta D\;\cdot \;0.001+1\right)}{1+\text{AR}\left(\delta D\;\cdot \;0.001+1\right)}\right]$$

AR represents the absolute ratio constant for deuterium based on the Vienna Standard Mean Ocean Water (VSMOW) standard and equates to 0.00015595 [29].

### Immunoblotting

Muscle biopsies were collected during basal conditions (biopsy 3) and 1 h postfeeding (biopsy 1, following breakfast containing a suboptimal dose of protein; biopsy 2, following the same breakfast plus the supplement) to investigate the acute anabolic signaling events involved in regulating muscle protein synthesis. Following homogenization of muscle for integrated MPS analysis, the total protein concentration of the sarcoplasmic fraction was determined by using a bicinchoninic acid assay (ThermoFisher Scientific), and this was used for immunoblotting. The protein concentration of the supernatants was mixed with 4× Laemmli buffer containing 10% 2-mercaptoethanol and distilled water to achieve a final protein concentration of 2 μg/μL. On a 26-well 4%–15% TGX Stain-Free Precast Gels (Bio-Rad), 5 μL (10 μg) of each sample was loaded into wells with a protein ladder (Precision Plus All Blue Protein Standard; Bio-Rad) and a 4-point internal standard calibration curve (2.5, 5, 7.5, and 10 μL) comprised a pooled sample. Gel electrophoresis was performed at 200 V for 45 min at room temperature. After UV activation of the gels, the proteins were transferred to a nitrocellulose membrane by turbo transfer.

Membranes were cut accordingly to assess multiple proteins on a single membrane and blocked with 5% bovine serum albumin for 90 min at room temperature. Following blocking, membranes were incubated at 4°C in primary antibodies (1:1000) overnight (Cell Signaling Technology); total form: mechanistic target of rapamycin complex 1 (mTOR) (#2972S), protein kinase B (AKT) (#4691S), ribosomal protein (rp) S6 (#2217S), and eukaryotic translation initiation factor 4E-binding protein 1 (4E-BP)1 (#9644S); phosphorylated form: mTOR^Ser2448^ (#5536S), AKT^Thr308^ (#13038S), rpS6^Ser235/236^ (#2211S), and 4E-BP1^Thr37/46^ (#2855S). Membranes were washed 3 × 10 min in Tris-buffered saline and Tween 20 (TBS-T; Millipore) and incubated with secondary antibodies (anti-rabbit IgG, 7074S; Cell Signaling Technology) for 90 min at room temperature and washed again. Proteins were detected by chemiluminescence (Clarity Max Western ECL substrate; Bio-Rad) and ChemiDoc MP Imaging system. Data were analyzed using Image Lab Software 6.0.1. and presented as the ratio of phosphorylated to total (P/T) protein.

### Blood analyses

Blood samples were collected in EDTA-containing and SST-containing vacutainers (Becton, Dickinson and Company) for plasma and serum, respectively. Following collection, blood samples were inverted, allowed to clot, centrifuged, and stored according to the manufacturer’s instructions. Plasma and serum were aliquoted into Eppendorf tubes for later analysis. Plasma glucose was assessed using the CONTOUR NEXT ONE blood glucose meter (Ascensia Diabetes Care Canada), and serum insulin was assessed by UV-Visible Spectrophotometer-CHEM7 by McMaster University core clinical laboratory services.

Plasma AA analysis was done using phenylthiocarbamyl AAs and high-performance liquid chromatography. This analysis was performed at SickKids, Toronto, Canada, at the SickKids Proteomics, Analytics, Robotics, and Chemical Biology Centre (SPARC https://lab.research.sickkids.ca/sparc/; SickKids Hospital, Toronto, ON). Briefly, plasma samples were dried and then hydrolyzed using 6 N HCl with 1% phenol at 110°C for 24 h under prepurified nitrogen. Following hydrolysis, samples were derivatized with phenyisothiocyanate to produce phenylthiocarbamyl AAs and analyzed using reverse-phase high-performance liquid chromatography. Data are presented as plasma leucine, BCAA, EAAs, and total AAs (excluding aspartic acid and glutamine).

### Statistical analysis

Participant baseline characteristics were assessed using a 1-way analysis of variance (ANOVA). The majority of outcomes (muscle protein synthesis, anabolic signaling, blood analytes, and physical activity) were analyzed using a 2-way, mixed-design ANOVA, with time (control compared with supplemental phase) as the within-subject factor and group (pea compared with whey compared with collagen group) as the between-subject factor. Dietary analysis was performed using a mixed-design ANOVA, with time (habitual compared with control compared with supplemental phase) as the within-subject factor and group (pea compared with whey compared with collagen group) as the between-subject factor. Significance was set at *P* < 0.05. When a significant interaction effect was identified, a Bonferroni post hoc correction was applied to adjust for multiple comparisons. Data are presented as means ± SD unless otherwise indicated. Data are plotted as box and whiskers plots where the whiskers represent maximum and minimum values, the box the IQR (25%–75%), the line in the box the median, and the plus sign the mean. All data analyses were performed using IBM SPSS Statistics for Mac, version 28.0.

## Results

### Participants

**FIGURE 2:**
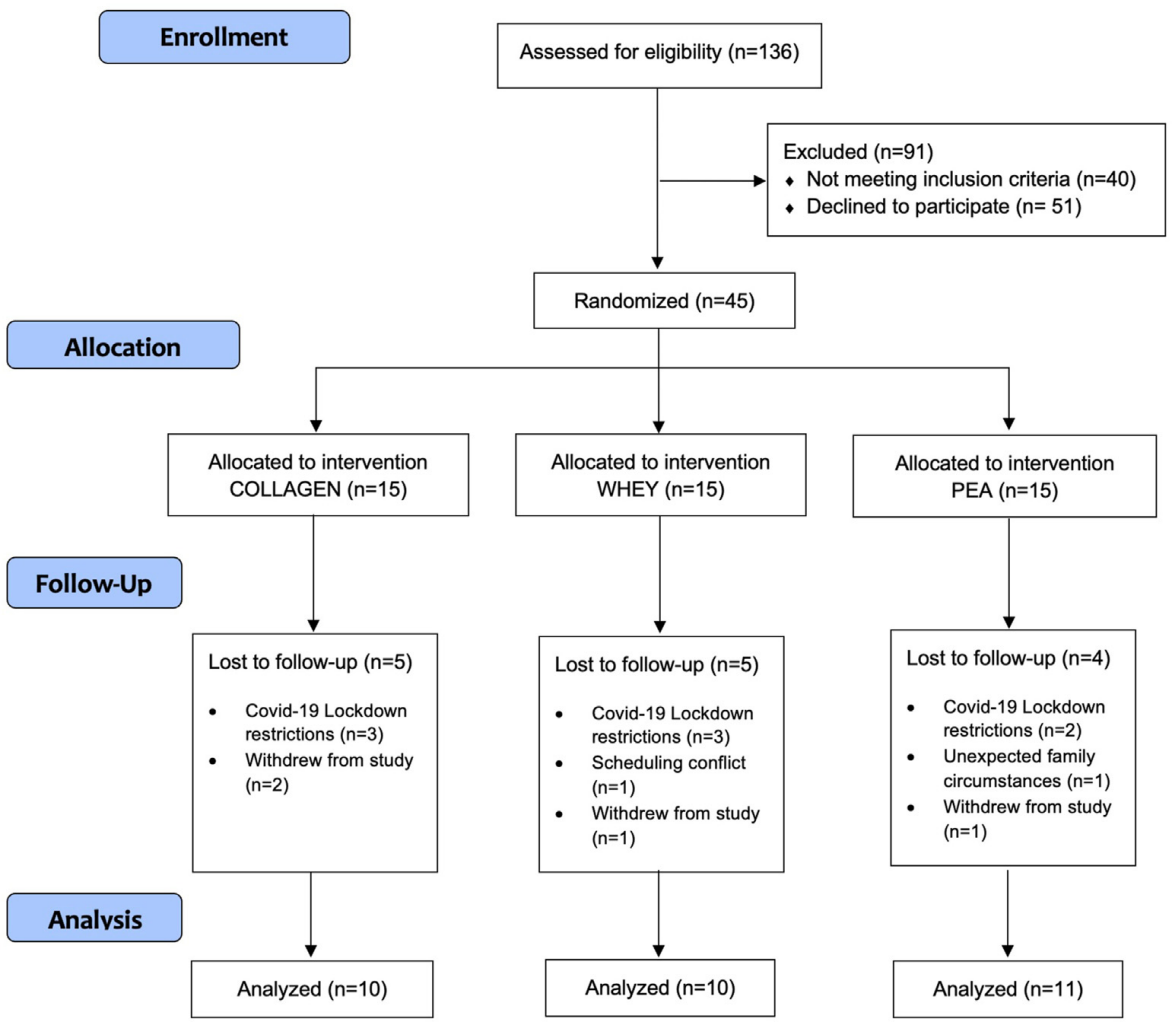
CONSORT participant flow diagram.

One hundred and thirty-six older males were contacted and assessed for eligibility; 91 were excluded at this stage because they did not meet the inclusion criteria or declined to participate. Forty-five individuals were randomly assigned to 1 of the 3 intervention groups: whey (*n* = 15), pea (*n* = 15), or collagen (*n* = 15). Fourteen individuals did not complete the study (whey; *n* = 5, pea; *n* = 4, and collagen; *n* = 5) for reasons unrelated to the trial. Details are contained in Figure 2. Participant characteristics are presented in Table 2.

**TABLE 2:** Participant’s anthropometric and physical function characteristics

	Collagen (*n* = 10)	Whey (*n* = 10)	Pea (*n* = 11)
Age (y)	72 ± 4	72 ± 5	72 ± 5
Height (m)	1.76 ± 0.05	1.78 ± 0.05	1.76 ± 0.07
Body mass (kg)	85.7 ± 12.4	83.5 ± 10.4	79.2 ± 10.4
BMI (kg/m^2^)	27.7 ± 3.7	26.4 ± 2.4	25.4 ± 2.5
Total fat mass (kg)	25.3 ± 8.1	24.8 ± 6.4	22.5 ± 6.1
Body fat (%)	30.3 ± 6.2	30.6 ± 5.2	29.6 ± 5.74
Total lean mass (kg)	56.7 ± 6.1	55.2 ± 5.6	53.0 ± 5.9
Appendicular lean mass (kg)	26.8 ± 3.5	25.1 ± 2.67	24.4 ± 3.1
Appendicular lean mass/BMI	0.97 ± 0.07	0.95 ± 0.08	0.97 ± 0.10
Handgrip strength (kg)	40.6 ± 8.3	45.4 ± 6.4	41.7 ± 4.4
Handgrip strength/BMI (kg)	1.49 ± 0.36	1.73 ± 0.26	1.65 ± 0.17
6-min walk (s)	4.84 ± 0.64	4.81 ± 0.83	5.14 ± 0.81
Gait speed (m/s)	1.26 ± 0.17	1.28 ± 0.25	1.19 ± 0.18
Fasting glucose (mmol/L)	6.0± 0.6	5.6 ± 0.5	5.5 ± 0.4
Fasting insulin (pmol/L)	56.7 ± 38.2	50.9 ± 17.4	65.4 ± 37.0

Abbreviation: ANOVA, analysis of variance; BMI, body mass index.

Statistical analysis was performed with a 1-way ANOVA. Values are means ± SD.

### Dietary intake

The composition of participants’ habitual dietary intake and the diet consumed during study involvement can be found in Table 3. Three participants did not complete the dietary records provided; the habitual diet data presented are for collagen (*n* = 8), whey (*n* = 10) and pea (*n* = 10). No significant interaction effects were observed for any comparison. A significant main effect of time was observed for absolute protein intake, with the control phase significantly lower than the habitual phase (*P* < 0.001) and the supplemental phase significantly greater than the habitual (*P* = 0.042) and control (*P* < 0.001) phases. A significant main effect of time was also observed for relative protein intake, with the control phase significantly lower than the habitual phase (*P* < 0.001) and the supplemental phase (*P* < 0.001), and no difference between supplemental and habitual phases (*P* = 0.088).

**TABLE 3:** Energy and macronutrient composition of participant’s habitual diets and intake during the separate study phases

	Habitual diet	Control phase	Supplemental phase
Collagen			
*n*	8	10	10
Energy (kcal)	2359 ± 576	2214 ± 206	2410 ± 206
Carbohydrate (g/d)	272 ± 73	307 ± 27	310 ± 27
Fat (g/d)	91 ± 43	83 ± 8	83 ± 8
Protein (g/d)	91 ± 23^a^	70 ± 9^b^	112 ± 9^c^
Protein/body mass (g/kg)	1.08 ± 0.32^a^	0.81 ± 0.01^b^	1.32 ± 0.08^a^
Whey			
*n*	10	10	10
Energy (kcal)	2330 ± 509	2201 ± 244	2402 ± 244
Carbohydrate (g/d)	293 ± 95	307 ± 31	310 ± 31
Fat (g/d)	90 ± 30	81 ± 12	84 ± 12
Protein (g/d)	95 ± 21^a^	68 ± 7^b^	108 ± 7^c^
Protein/body mass (g/kg)	1.16 ± 0.36^a^	0.82 ± 0.02^b^	1.30 ± 0.08^a^
Pea			
*n*	10	11	11
Energy (kcal)	2433 ± 835	2108 ± 236	2308 ± 236
Carbohydrate (g/d)	298 ± 122	296 ± 32	296 ± 31
Fat (g/d)	92 ± 32	78 ± 10	83 ± 10
Protein (g/d)	91 ± 34^a^	64 ± 8^b^	104 ± 8^c^
Protein/body mass (g/kg)	1.21 ± 0.50^a^	0.81 ± 0.01^b^	1.32 ± 0.07^a^

Abbreviation: ANOVA, analysis of variance.

Statistical analysis was performed with a mixed-design ANOVA, with time (habitual vs. control vs. supplemental) as the within-subject factor and group (pea vs. whey vs. collagen) as the between-subject factor. The supplemental phase energy and macronutrient composition includes the 50 g of additional protein consumed daily as part of the intervention. Dissimilar letters indicate a significant main effect of time. Values are means ± SD.

### Physical activity

Participants’ physical activity data are presented in Table 4. No significant differences in physical activity metrics were observed within or between groups.

**TABLE 4:** Accelerometer-derived physical activity data during study phases

	Control phase	Supplemental phase
Collagen		
*n*	10	10
Wear time (%)	85 ± 14	85 ± 12
Step count (steps/d)	11168 ± 3118	11293 ± 2702
Sedentary activity (min) [%]	621 ± 176 [49.6 ± 8.6]	604 ± 178 [48.7 ± 9.7]
Light activity (min) [%]	451 ± 66 [37.7 ± 7.3]	450 ± 59 [37.9 ± 7.4]
Moderate activity (min) [%]	156 ± 46 [12.7 ± 3.1]	164 ± 55 [13.4 ± 4.1]
Whey		
*n*	10	10
Wear time (%)	81 ± 14	79 ± 15
Step count (steps/d)	11796 ± 3136	11223 ± 3296
Sedentary activity (min) [%]	590 ± 193 [49.6 ± 10.5]	577 ± 175 [49.4 ± 7.8]
Light activity (min) [%]	421 ± 73 [37.1 ± 8.1]	396 ± 95 [37.3 ± 5.9]
Moderate activity (min) [%]	147 ± 23 [13.3 ± 3.9]	140 ± 28 [13.0 ± 3.9]
Pea		
*n*	11	10
Wear time (%)	75 ± 12	72 ± 13
Step count (steps/d)	11,144 ± 4374	10,778 ± 4166
Sedentary activity (min) [%]	474 ± 178 [42.3 ± 11.2]	417 ± 187 [42.0 ± 13.0]
Light activity (min) [%]	446 ± 33 [43.0 ± 6.5]	437 ± 40 [43.9 ± 7.5]
Moderate activity (min) [%]	159 ± 89 [14.7 ± 8.8]	142 ± 94 [14.4 ± 9.5]

Abbreviation: ANOVA, analysis of variance.

Statistical analysis was performed with a mixed-design ANOVA, with time (control vs. supplemental) as the within-subject factor and group (pea vs. whey vs. collagen) as the between-subject factor. Values are means ± SD.

### Integrated MPS and body water ^2^H enrichment

Body water ^2^H enrichment increased (∼0.4-0.5 APE) 24 h after D_2_O loading and stable isotopic enrichment was maintained throughout the remainder of the study, with no significant difference between groups at any time point (Supplemental Figure 1).

**FIGURE 3:**
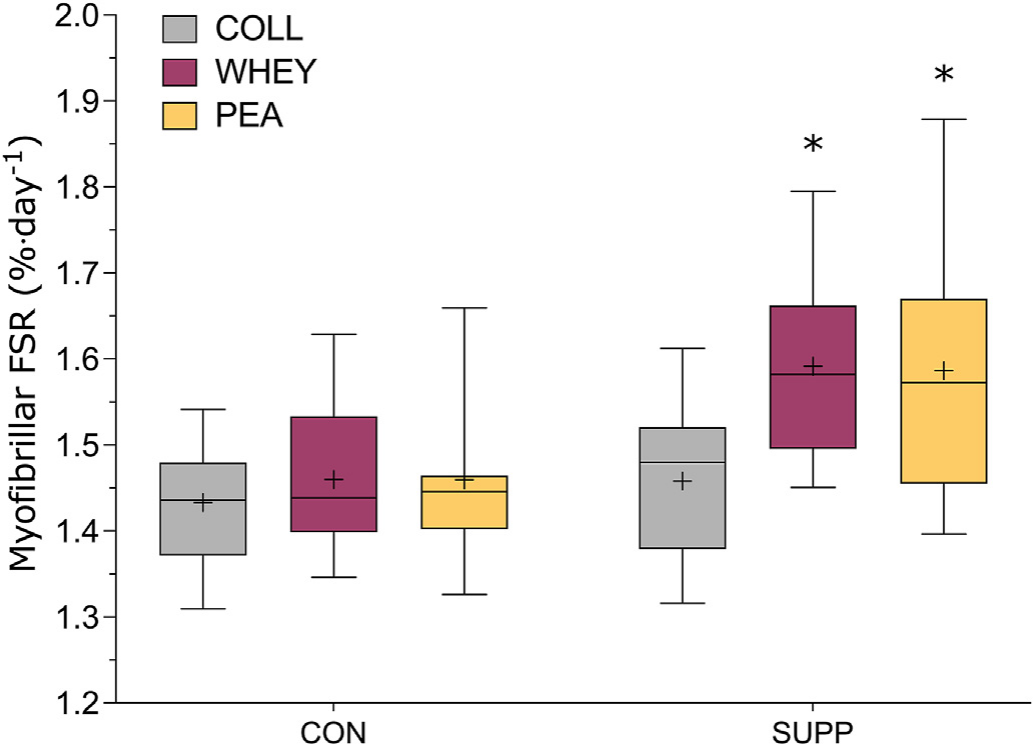
Integrated myofibrillar fractional synthetic rate (FSR) in healthy older males separated by group (collagen, *n* = 10; whey, *n* = 10; pea, *n* = 11) and study phase. Box and whisker plot showing the integrated myofibrillar FSR. The horizontal line within the boxes depicts the median of each group and the cross depicts the mean. The upper and lower edges of the box represent the 75th and 25th quartiles, respectively. The upper and lower whiskers represent the maximum and minimum values, respectively. CON represents the study phase where participants were fed protein at the RDA, and SUPP represents the study phase where participants were supplemented with 2 × 25 g of their randomly assigned protein supplement. ∗Significant difference from the control phase within the group (*P* < 0.05).

A significant group × time interaction effect was observed (*P* = 0.001), and pairwise comparisons revealed that integrated rates of MPS were ∼9% greater in the supplemental phase than those in the control phase for both whey (1.59 ± 0.11 %/d compared with 1.46 ± 0.09 %/d; *P* < 0.001; Hedges *g* = 1.33) and pea (1.59 ± 0.14 %/d compared with 1.46 ± 0.10%/d; *P* < 0.001, Hedges *g* = 1.04) groups (Figure 3). In contrast, integrated MPS did not significantly increase during the supplemental phase (1.46 ± 0.09%/d compared with 1.43 ± 0.07%/d; *P* = 0.237) in the collagen group. Pairwise comparisons also revealed that compared with the collagen group, whey (*P* = 0.052, Hedges *g* = 1.30) and pea (*P* = 0.057, Hedges *g* = 1.07) were trending toward greater MPS in the supplemental phase.

### Anabolic signaling

**FIGURE 4:**
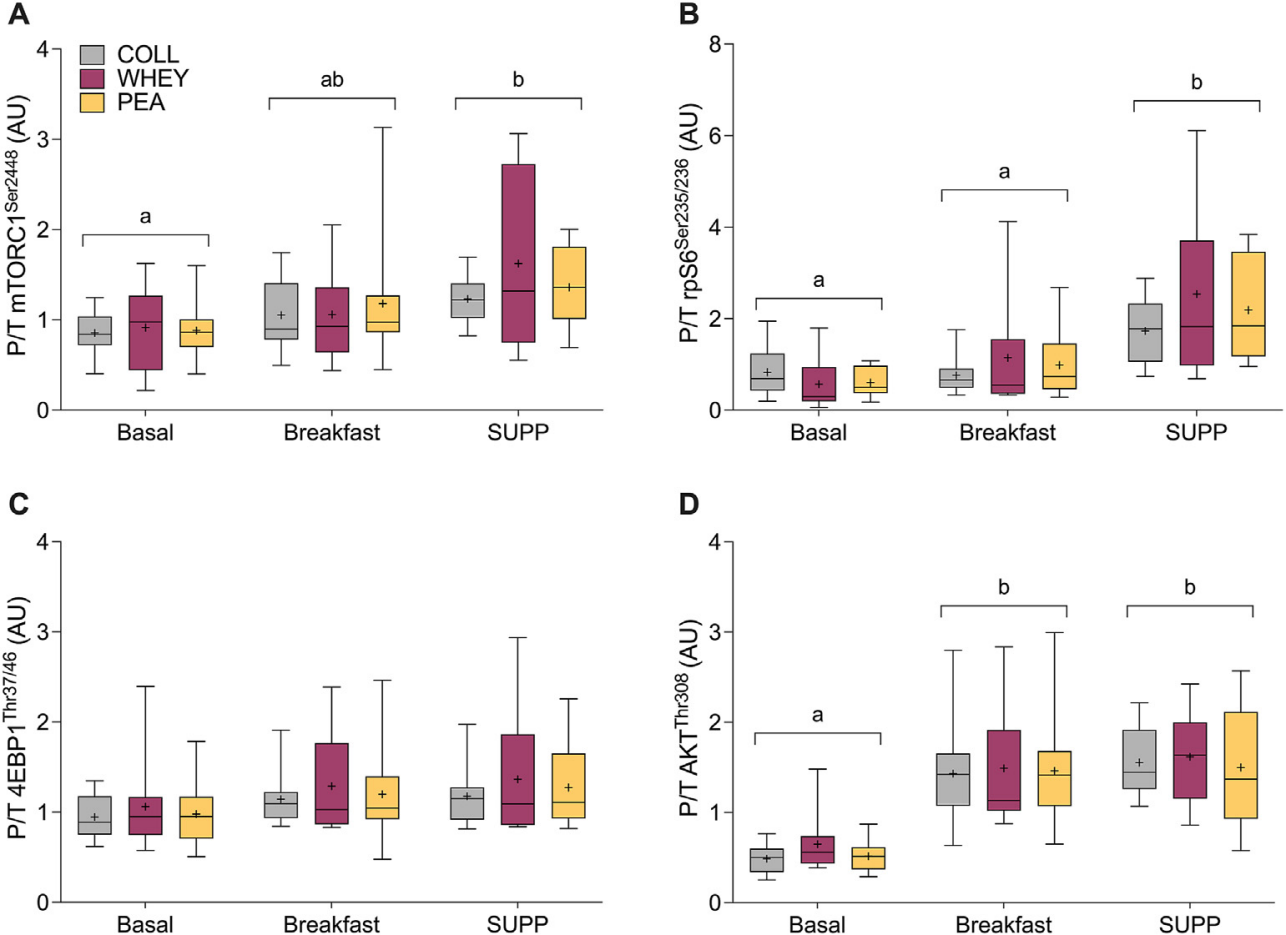
Anabolic signaling proteins involved in the mechanistic target of rapamycin complex (mTORC) 1 pathway are presented as the ratio of phosphorylated to total protein. The data included reflect all participants (collagen, *n* = 10; whey, *n* = 10; pea, *n* = 11), except for 1 missing sample during supplemental in pea (*n* = 10) across all included proteins, 3 missing values for ribosomal protein S6 (rpS6, *n* = 7), and 1 missing value for protein kinase B (AKT, *n* = 9) during breakfast-only in the whey group. (A) mTORC1; (B) rpS6; (C) 4E-BP1; (D), AKT. P/T = ratio of phosphorylated to total protein. No interaction effect (time × group) or effect of group (i.e., protein supplement) was observed. Dissimilar letters indicate a significant main effect of time.

The anabolic signaling response to breakfast and supplemental conditions can be found in Figure 4. No significant interaction effects were observed for the anabolic protein signaling events. A significant effect of time was observed for mechanistic target of rapamycin complex 1 (mTORC1^Ser2448^) with supplemental significantly greater than the basal condition (*P* < 0.001). A significant main effect of time was observed for rpS6^Ser235/236^, with the phosphorylation status during supplemental being significantly greater than basal (*P* < 0.001) and breakfast (*P* < 0.001). A significant main effect of time was also observed for AKT^Thr308^ with supplemental (*P* < 0.001) and breakfast (*P* < 0.001) greater than basal. No significant effects were observed for 4E-BP1^Thr37/46^. Representative blot images are shown in Supplemental Figure 2.

### Plasma AAs

**FIGURE 5:**
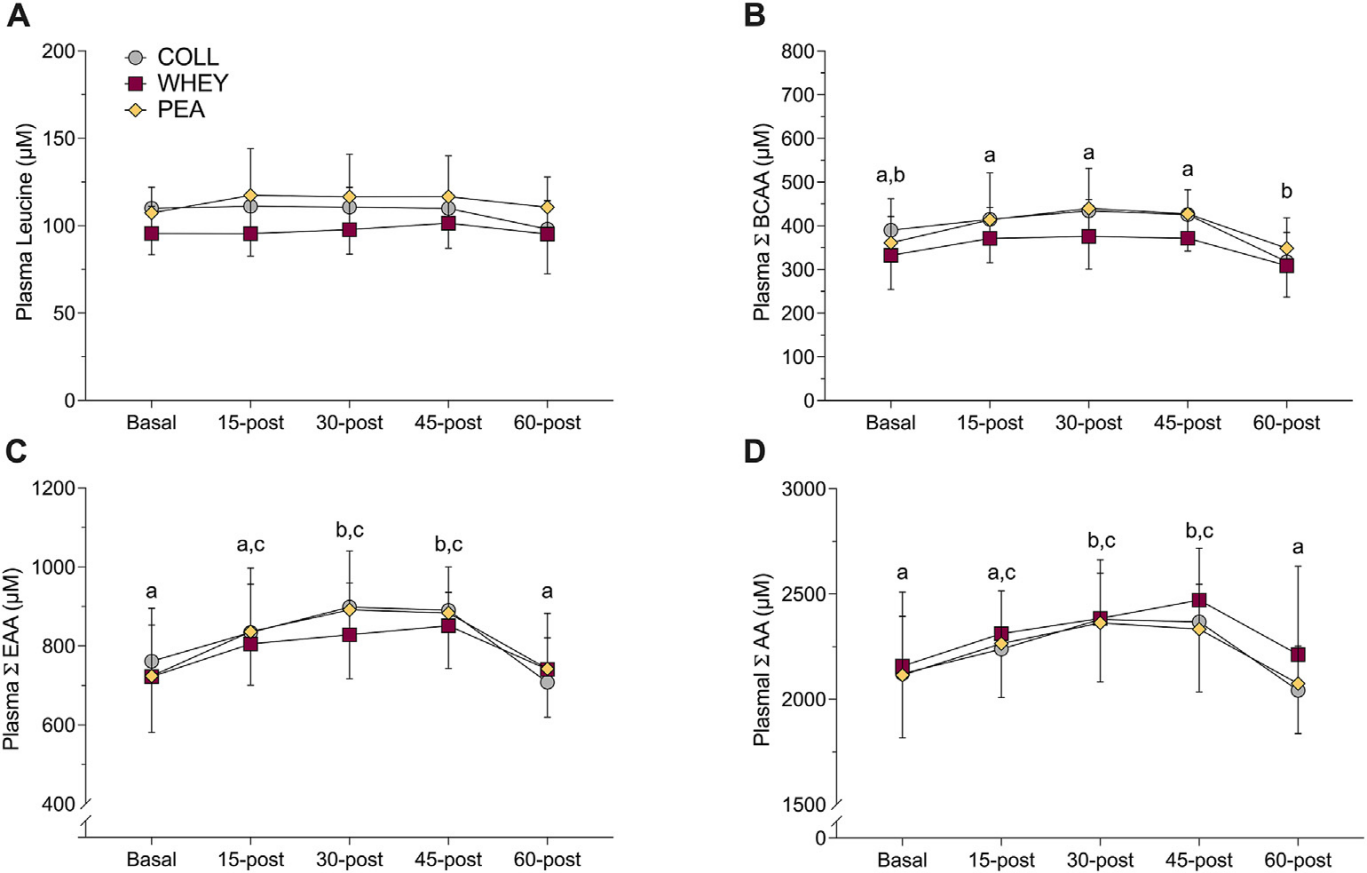
Plasma amino acid concentrations during the control (nonsupplemented) phase ≤60 min postingestion (collagen, *n* = 10; whey, *n* = 10; pea, *n* = 11) in the breakfast-only (visit 3) acute feeding trial. (A) Plasma leucine; (B) plasma branched-chain amino acids (BCAAs); (C) plasma essential amino acids (EAAs); and (D) plasma total amino acids (AAs; excluding aspartic acid and glutamine). No interaction effect (time × group) or effect of group (i.e., protein supplement) was observed. Dissimilar letters indicate a significant main effect of time. Values are expressed as means ± SD.

**FIGURE 6:**
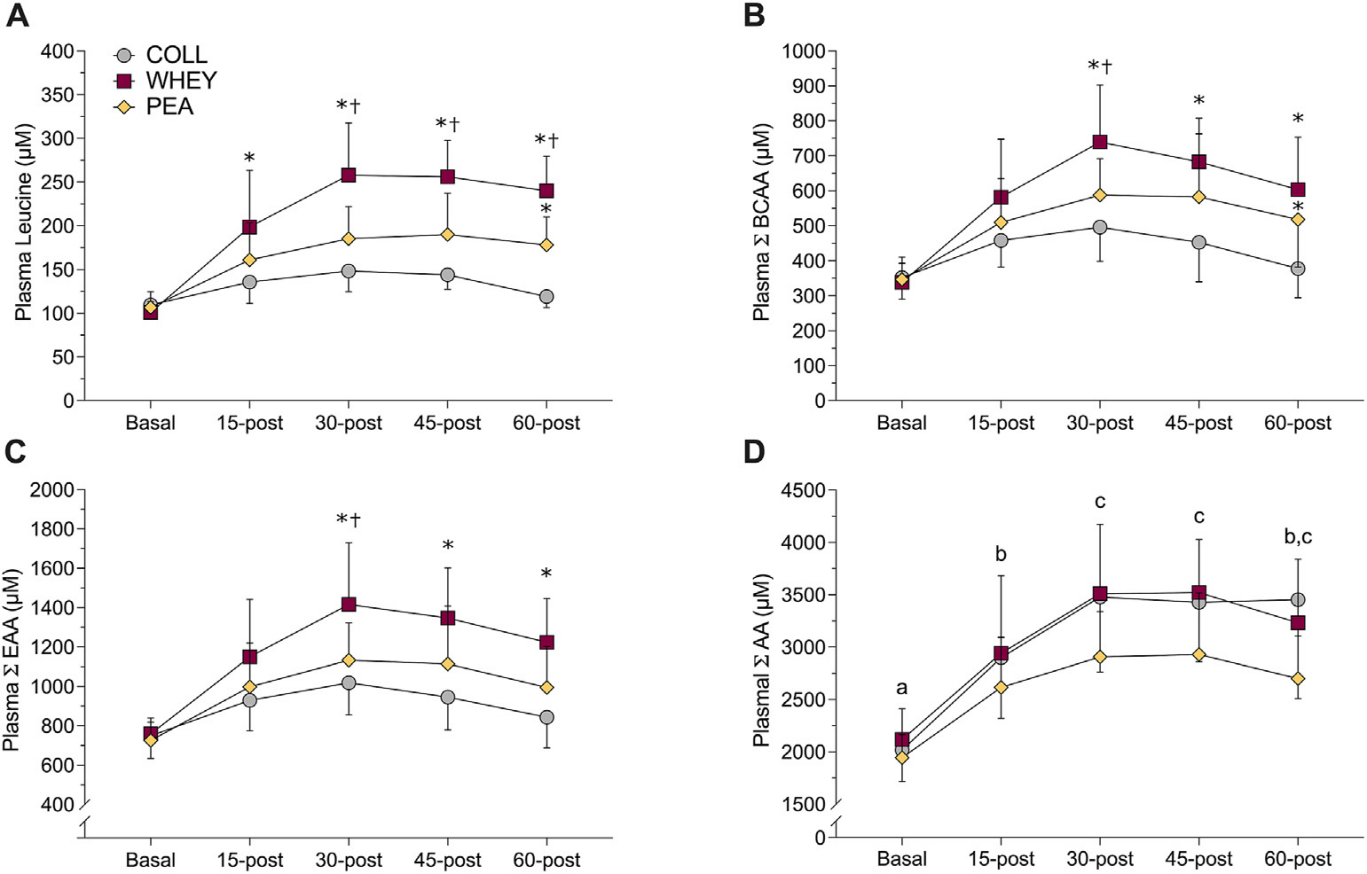
Plasma amino acid concentrations ≤60-min postingestion (collagen, *n* = 10; whey, *n* = 10; pea; *n* = 10; no visit 4 samples for 1 participant in the pea group) in the breakfast + supplemental (visit 4) acute feeding trial. (A) Plasma leucine; (B) plasma branched-chain amino acids (BCAAs); (C) plasma essential amino acids (EAAs), and (D) plasma total amino acids (AAs; excluding aspartic acid and glutamine). ∗Significantly different from collagen at that time point (*P* < 0.05). †Significantly different from pea at that time point. Dissimilar letters indicate a significant main effect of time. Values are expressed as means ± SD.

Plasma AA data following breakfast are presented in Figure 5. No significant interactions were observed for plasma leucine, BCAA, EAAs, or total AA during control (visit 3—breakfast-only). A significant effect of time was observed for plasma BCAA, EAA, and total AA concentrations, with no differences between the conditions. Plasma AA data following supplemental are presented in Figure 6. A significant interaction was observed for plasma leucine, BCAA, and EAA concentrations during supplemental phase (visit 4—breakfast plus supplement) (Figure 6). Whey group was significantly greater than collagen group at 15-min after and collagen and pea groups at 30-, 45-, and 60-min after for leucine. Whey group was significantly greater than collagen and pea groups at 30-min after and collagen at 45- and 60-min after for BCAAs. Whey group was significantly greater than collagen and pea groups at 30-min after and collagen at 45- and 60-min after for EAAs. Pea group was significantly greater than collagen at 60-min after for leucine and BCAAs. A main effect of time was observed for total AAs.

### Plasma glucose and serum insulin concentrations

No significant interaction effects were observed for plasma glucose concentration. A main effect of time for glucose and insulin were observed during the breakfast-only (control) and breakfast with supplement (supplemental) acute feeding trials (Supplemental Figure 3).

## Discussion

In accordance with our hypothesis, we discovered that consuming higher-quality isolated protein supplements such as whey and pea during the supplemental phase enhanced MPS rates compared with consuming protein at the RDA. Notably, the increases in MPS rates we observed with whey and pea during the supplemental phase occurred without an activity (loading) stimulus. In contrast, and inconsistent with our hypothesis, twice daily collagen protein supplementation (50 g total/d) did not increase integrated MPS rates above basal in older males; we propose that this is due to the low leucine content and overall protein quality of collagen. We also demonstrated that a whole-food breakfast containing a relatively low (∼15 g) protein content did not stimulate the mTORC1 signaling pathway and presumably MPS. However, providing additional protein (25 g/serving) led to increased phosphorylation of mTORC1 and rpS6. We showed that whey protein consumption led to the greatest postprandial plasma aminoacidemia. Despite this difference, integrated MPS rates were similar in the whey and pea conditions but remained unchanged in the collagen condition despite participants consuming an additional 50 g of collagen protein as peptides per day.

Current protein recommendations for older adults are insufficient to maintain skeletal muscle mass [14,15,32]. Muscle-related anabolic resistance in older individuals raises several questions about the sufficiency of the RDA for protein intake [33]. Despite a reanalysis of the original nitrogen balance data [11] showing protein requirements are higher than estimated [34], nitrogen balance data showing older persons require ∼30% more protein to balance intake [11], newer methods showing the inadequacy of the RDA [[34], [35], [36]], and experts advocating for an increased (1.0–1.2 g/kg/d) dietary protein recommendation [[14], [15], [16]], the RDA remains 0.8 g/kg/d, regardless of age [11]. We provide evidence that MPS is lower when older males consume protein at the RDA. We showed that increasing daily protein intake by ∼60% from the RDA level to ∼1.3 g/kg/d during the supplemental phase, much closer to suggested protein intakes for older adults [[14], [15], [16]], augmented integrated MPS rates by ∼10%. These data align with previous studies demonstrating that increasing the total daily protein intake of older individuals led to a more positive whole-body net protein balance [37], greater synthetic rates of glutathione [38], and greater retention of skeletal muscle [39].

Plant-derived proteins are chosen as foods for several reasons [40,41]. However, plant-derived proteins are generally considered lower-quality—reflected in lower protein digestibility-corrected AA and digestible indispensable AA scores [42]—than animal-based protein sources owing to reduced digestibility and insufficient quantities of EAAs, particularly leucine [18]. Plant-protein isolates provide a digestible protein source, of which the AA composition can be modified to provide lower than recommended EAAs [41]. In our study, we fed older males 25 g of protein twice daily, with each serving of whey and pea protein-containing twice the amount of EAAs and 140% (1.68 g) and 131% (1.62 g) more leucine, respectively, compared with collagen (0.70 g). Combining the protein supplement (whey and pea) with the breakfast and lunch meals likely surpassed the muscle-specific leucine threshold necessary to switch on the protein synthetic machinery, containing the EAAs needed to maintain a heightened MPS response to feeding [[43], [44], [45], [46]]. However, the limited EAA, particularly leucine, content in the collagen supplement was likely insufficient to initiate an acute increase in MPS, which, when repeated, led to lower rates of integrated MPS. Our data align with recent studies in younger individuals demonstrating the effectiveness of plant-derived protein sources in stimulating MPS [47,48] and supporting equivalent muscle hypertrophy exercise training [49,50].

We have shown that collagen protein contains insufficient EAAs and leucine to stimulate and maintain a robust increase in MPS in older males [51,52]. Others have used collagen supplements and observed no benefits in stimulating muscle collagen synthesis [53]. No acute or chronic data show that collagen can stimulate MPS in younger or older persons. Nonetheless, collagen supplementation in combination with resistance training in older sarcopenic males has been reported to have extraordinary benefits on body composition [54]; however, this finding remains inadequately explained [55].

We have postulated that suboptimal stimulation of MPS is a major driver of skeletal muscle loss in older individuals [56,57]. Typically, younger individuals require a modest per-meal dose (0.24 g/kg/meal) of protein (and likely leucine) to saturate the feeding-induced rise in MPS [8], whereas older individuals require a much larger (0.4 g/kg/meal) bolus [9]. In this study, we have shown that feeding a breakfast containing a relatively low amount of protein (∼15 g) did not significantly increase the phosphorylation of proteins implicated in the mTORC1 signaling pathway (mTORC1 and rpS6). This finding may be unsurprising as we observed no feeding-induced changes in plasma leucine and only a slight rise in plasma EAAs and total AAs [58]. Conversely, when older males were fed a protein supplement with their breakfast (and lunch) meal, yielding a per-meal protein dose of ∼40 g (∼0.5 g/kg), we observed an increase in the phosphorylation of mTORC1 and rpS6, and these data corroborate the hyperaminoacidemia following the breakfast and supplement. Interestingly, the acute anabolic signaling events were upregulated irrespective of the protein supplement provided. Acute signaling (e.g., phosphorylation) events provide a snapshot at a single time point and are afflicted with substantial variability within and between individuals. Thus, although subtle differences in aminoacidemia are observed, discrepancies among acute protein signaling events and rates of MPS have been demonstrated previously and, as such, are inappropriate as a proxy of skeletal muscle anabolism [8,59,60].

Our data support the idea that providing supplemental protein to older males is efficacious in overcoming the acute protein signaling deficits observed in muscle anabolic resistance [61]. A recommendation for redistributing protein intake from the largest protein-containing meal, usually the evening meal in older persons [62,63], to a more evenly spaced pattern of protein feeding with each meal containing sufficient protein to maximize the MPS in older persons may be warranted; longer-term observational data support such a practice [28,64,65]. However, maximizing and maintaining the MPS response to protein feeding and repeating this throughout the day is reliant on the protein source providing sufficient EAAs and leucine, and collagen protein lacks the necessary substrates to augment muscle anabolism, so may not be beneficial for retaining muscle mass in older individuals.

In this study, we addressed age-related muscle anabolic resistance with an ostensibly optimal protein feeding approach—manipulating total daily protein intake, protein quality, and distribution—and this led to an enhanced rate of integrated MPS in older males. However, our study is not without limitations. We recognize that skeletal muscle mass is the product of chronic changes in net protein balance—the algebraic difference between MPS and MPB—and while we did not assess MPB due to the technical difficulties of obtaining accurate measures [66], we know that MPB fluctuates far less than MPS. For example, MPB remains unchanged with aging [67] and muscle disuse [60] and increases with resistance training to facilitate remodeling [6]. Therefore, increasing protein intake at breakfast and lunch may promote long-term muscle growth primarily by influencing MPS. Another limitation of this study is that we did not recruit older female participants, which hampers the generalizability of the study’s findings. Although it may be unlikely that older males and females would display divergent responses to high-quality animal and plant-based protein supplementation beyond the RDA, this remains to be confirmed.

In conclusion, we discovered that the RDA was insufficient to support higher rates of MPS in older adults. Manipulating dietary protein to increase daily consumption of higher-quality—whey and pea but not collagen—proteins by targeting the lowest protein-containing meals offers a viable strategy to enhance integrated MPS in older adults. Consuming protein much closer to expert group consensus recommendations [[14], [15], [16]] may help to increase integrated MPS, preserve skeletal muscle mass with advancing age, and extend healthspan—compressing the years of disease and disability commonly experienced by older individuals closer to the end of life.

We realize that resistance exercise works synergistically with high-quality protein nutrition to augment MPS further; therefore, future research efforts could seek to understand the longer-term impact of plant-derived protein supplementation in combination with increased physical activity (e.g., resistance training) in older males and females on skeletal muscle mass and physical function.

## Author contributions

The authors responsibilities were as follows – JM, SMP: designed the research (project conception, development of overall research plan, and study oversight); JM, CVL, AN, MM, CL, BSC, SMP: conducted research; JM, CVL, AN, CL, SMP: analyzed data or performed statistical analysis; JM, CVL, AN, SMP: wrote the article; JM, SMP: had primary responsibility for the final content; and all authors: have read and approved the final manuscript.

### Conflict of interest

JM, CVL, AN, MM, CL, and BSC declared no conflicts of interest. SMP reports grants or research contracts from the United States National Dairy Council, Canadian Institutes for Health Research, Cargill, Friesland Campina, Dairy Farmers of Canada, Ontario Centre of Innovation, Nestle Health Sciences, Myos, National Science and Engineering Research Council, and the United States NIH during the conduct of the study; personal fees from Nestle Health Sciences; and nonfinancial support from Enhanced Recovery, outside the submitted work. SMP has patents licensed to Exerkine but reports no financial gains from patents or related work. The funders had no role in the study design, data collection, analyses, or interpretation of data; in the writing of the manuscript; or in the decision to publish the results.

### Funding

This study was supported by a research contract from Roquette Frères. JM and CL were supported by postdoctoral fellowships from the Canadian Institute of Health Research (CIHR). BSC was supported by a Canadian Graduate Scholarship from the Natural Sciences and Engineering Research Council of Canada (NSERC).

### Data availability

Data described in the manuscript will be made available on request pending the filing of any IP and patent claims and approval from the corresponding author (SMP).
