# Supplementary material for: The effects of whey, pea, and collagen protein supplementation beyond the recommended dietary allowance on integrated myofibrillar protein synthetic rates in older males: a randomized controlled trial
Source: Am J Clin Nutr. 2024 May 16;120(1):34–46. doi: 10.1016/j.ajcnut.2024.05.009 (PMC11291473; doi:10.1016/j.ajcnut.2024.05.009)
Supplement: Multimedia component 1 [file mmc1.docx]

**Supplementary Figure 1.** ^2^H body water enrichment. APE; Atom percent excess. Values are means ± SD.

**Supplementary Figure 2.** Representative western blot images. C; COLL, W; WHEY, and P; PEA.


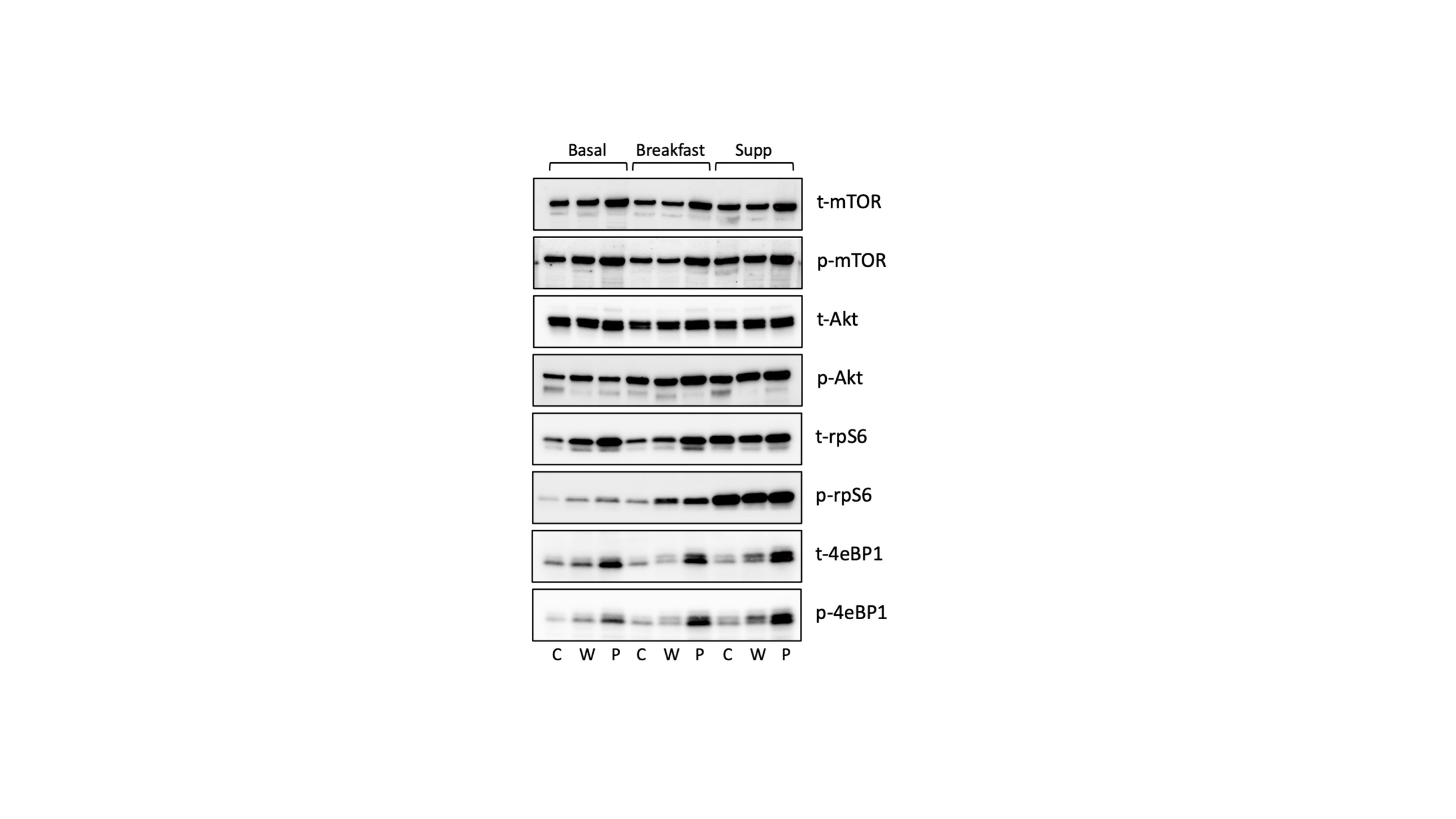


**Supplementary Figure 3.** Plasma glucose and serum insulin concentrations.

Panel **A**; plasma glucose and Panel **B;** serum insulin in response to CON (breakfast-only); Panel **C;** plasma glucose and Panel **D;** serum insulin in response to SUPP (breakfast plus supplement). No interaction effect (time x group) or effect of group (i.e., protein supplement) was observed. Dissimilar letters indicate a significant main effect of time. Values are expressed as means ± SD.
